# Supplementary material for: Rheological transition driven by matrix makes cancer spheroids resilient under confinement
Source: Life Sci Alliance. 2025 Mar 27;8(6):e202402601. doi: 10.26508/lsa.202402601 (PMC11949850; doi:10.26508/lsa.202402601)
Supplement: Supplementary file 1 [file LSA-2024-02601_Supplemental_Data_1.pdf]

Elastic Modulus

Number of families	1
Number of comparisons per family	3
Alpha	0.05

Tukey's multiple comparisons test	Mean Diff.	95.00% CI	Significant?	Summary	Adjusted P Value	
moruloid vs. blastuloid	-213.6	-364.3 to -6	Yes	**	0.0057	A-B
moruloid vs. blastuloid -ECM	4.499	-128.3 to 1	No	ns	0.9958	A-C
blastuloid vs. blastuloid -ECM	218.1	95.16 to 34	Yes	***	0.0009	B-C

Test details	Mean 1	Mean 2	Mean Diff.	SE of diff.	n1	n2
moruloid vs. blastuloid	342.1	555.8	-213.6	58.37	4	5
moruloid vs. blastuloid -ECM	342.1	337.6	4.499	51.48	4	10
blastuloid vs. blastuloid -ECM	555.8	337.6	218.1	47.66	5	10

Aspect ratio slopes

Number of families	1
Number of comparisons per family	3
Alpha	0.05

Tukey's multiple comparisons test	Mean Diff.	95.00% CI	Significant?	Summary	Adjusted P Value	
moruloid vs. blastuloid	0.4005	0.2554 to 0	Yes	****	<0.0001	A-B
moruloid vs. blastuloid-ECM	0.03846	-0.1032 to (	No	ns	0.7784	A-C
blastuloid vs. blastuloid-ECM	-0.3621	-0.4993 to	Yes	****	<0.0001	B-C

Test details	Mean 1	Mean 2	Mean Diff.	SE of diff.	n1	n2
moruloid vs. blastuloid	0.3389	-0.06162	0.4005	0.05811	8	9
moruloid vs. blastuloid-ECM	0.3389	0.3005	0.03846	0.05673	8	10
blastuloid vs. blastuloid-ECM	-0.06162	0.3005	-0.3621	0.05495	9	10

Minor axis relaxation time

Number of families	1
Number of comparisons per family	3
Alpha	0.05

Tukey's multiple comparisons test	Mean Diff.	95.00% CI	Significant?	Summary	Adjusted P Value	
moruloid vs. blastuloid	-0.2358	-0.4523 to	Yes	*	0.035	A-B
moruloid vs. blastuloid - ECM	0.004167	-0.2123 to (	No	ns	0.9982	A-C
blastuloid vs. blastuloid - ECM	0.24	0.008626 t	Yes	*	0.0431	B-C

Test details	Mean 1	Mean 2	Mean Diff.	SE of diff.	n1	n2
moruloid vs. blastuloid	0.7375	0.9733	-0.2358	0.07349	4	3
moruloid vs. blastuloid - ECM	0.7375	0.7333	0.004167	0.07349	4	3
blastuloid vs. blastuloid - ECM	0.9733	0.7333	0.24	0.07856	3	3

minor axis peak time

Number of families	1
Number of comparisons per family	3

Alpha	0.05

Tukey's multiple comparisons test	Mean Diff.	95.00% CI	Significant?	Summary	Adjusted P Value	
moruloid vs. blastuloid	3.329	-0.3134 to (	No	ns	0.0694	A-B
moruloid vs. blastuloid - ECM	-1.38	-5.022 to 2.	No	ns	0.515	A-C
blastuloid vs. blastuloid - ECM	-4.708	-8.350 to -1	Yes	*	0.0174	B-C

Test details	Mean 1	Mean 2	Mean Diff.	SE of diff.	n1	n2
moruloid vs. blastuloid	4.835	1.507	3.329	1.187	3	3
moruloid vs. blastuloid - ECM	4.835	6.215	-1.38	1.187	3	3
blastuloid vs. blastuloid - ECM	1.507	6.215	-4.708	1.187	3	3

Mean flow angle

Number of families	1
Number of comparisons per family	3
Alpha	0.05

Tukey's multiple comparisons test	Mean Diff.	95.00% CI	Significant?	Summary	Adjusted P Value	
moruloid vs. blastuloid	29.88	2.720 to 57	Yes	*	0.0279	A-B
moruloid vs. blastuloid - ECM	-5.447	-34.78 to 2	No	ns	0.8955	A-C
blastuloid vs. blastuloid - ECM	-35.33	-64.66 to -5	Yes	*	0.0146	B-C

Test details	Mean 1	Mean 2	Mean Diff.	SE of diff.	n1	n2
moruloid vs. blastuloid	41.4	11.52	29.88	11.26	20	20
moruloid vs. blastuloid - ECM	41.4	46.85	-5.447	12.16	20	15
blastuloid vs. blastuloid - ECM	11.52	46.85	-35.33	12.16	20	15

Spheroid damage

Number of families	1
Number of comparisons per family	3
Alpha	0.05

Tukey's multiple comparisons test	Mean Diff.	95.00% CI	Significant?	Summary	Adjusted P Value	
moruloid vs. blastuloid	19.24	0.5251 to 3	Yes	*	0.0444	A-B
moruloid vs. blastuloid - ECM	-5.363	-25.58 to 1	No	ns	0.7375	A-C
blastuloid vs. blastuloid - ECM	-24.6	-44.82 to -4	Yes	*	0.0203	B-C

Test details	Mean 1	Mean 2	Mean Diff.	SE of diff.	n1	n2
moruloid vs. blastuloid	24.69	5.45	19.24	6.55	4	4
moruloid vs. blastuloid - ECM	24.69	30.05	-5.363	7.074	4	3
blastuloid vs. blastuloid - ECM	5.45	30.05	-24.6	7.074	4	3

Area spheroid damage

Number of families	1
Number of comparisons per family	3
Alpha	0.05

Tukey's multiple comparisons test	Mean Diff.	95.00% CI	Significant?	Summary	Adjusted P Value

Moruloid vs. Blastuloid	-9684	-17978 to -	Yes	*	0.0185	A-B
Moruloid vs. Blastuloid - ECM	12410	5653 to 19	Yes	***	0.0001	A-C
Blastuloid vs. Blastuloid - ECM	22094	14204 to 2	Yes	****	<0.0001	B-C

Test details	Mean 1	Mean 2	Mean Diff.	SE of diff.	n1	n2
Moruloid vs. Blastuloid	18159	27843	-9684	3434	18	11
Moruloid vs. Blastuloid - ECM	18159	5749	12410	2797	18	24
Blastuloid vs. Blastuloid - ECM	27843	5749	22094	3267	11	24

**Aspect ratio**	**Ecadherin KD**
Table Analyzed	ctrl Ecadh KD
Column B	E-Cadherin KD
vs.	vs.
Column A	control
Unpaired t test	
P value	0.0008
P value summary	***
Significantly different (P < 0.05)?	Yes
One- or two-tailed P value?	Two-tailed
t, df	t=4.460, df=12
How big is the difference?	
Mean of column A	-0.0655
Mean of column B	0.3979
Difference between means (B - A) :	0.4634 ± 0.1039
95% confidence interval	0.2370 to 0.6897
R squared (eta squared)	0.6237
F test to compare variances	
F, DFn, Dfd	8.836, 7, 5
P value	0.029
P value summary	*
Significantly different (P < 0.05)?	Yes
Data analyzed	
Sample size, column A	6
Sample size, column B	8

**Apect Ratio**	**E-cadherin OE**
Table Analyzed	pcdh OE
Column B	E-cadherin OE
vs.	vs.
Column A	control
Unpaired t test	

P value	0.8839
P value summary	ns
Significantly different (P < 0.05)?	No
One- or two-tailed P value?	Two-tailed
t, df	t=0.1524, df=6
How big is the difference?	
Mean of column A	0.369
Mean of column B	0.3378
Difference between means (B - A) :	-0.03125 ± 0.2051
95% confidence interval	-0.5331 to 0.4706
R squared (eta squared)	0.003854
F test to compare variances	
F, DFn, Dfd	1.472, 3, 3
P value	0.7584
P value summary	ns
Significantly different (P < 0.05)?	No
Data analyzed	
Sample size, column A	4
Sample size, column B	4

E-cadherin KD mean flow angle

Table Analyzed	Data 1
Column B	E cadherin KD
vs.	vs.
Column A	control
Unpaired t test	
P value	0.0113
P value summary	*
Significantly different (P < 0.05)?	Yes
One- or two-tailed P value?	Two-tailed
t, df	t=2.727, df=26
How big is the difference?	
Mean of column A	21.45
Mean of column B	72.56
Difference between means (B - A) :	51.11 ± 18.74
95% confidence interval	12.59 to 89.63
R squared (eta squared)	0.2224
F test to compare variances	
F, DFn, Dfd	6.495, 13, 13
P value	0.0019
P value summary	**

Significantly different (P < 0.05)?	Yes
Data analyzed	
Sample size, column A	14
Sample size, column B	14

E-cadherin OE mean flow angle

Table Analyzed	Data 1
Column B	E-cadherin OE
vs.	vs.
Column A	control
Unpaired t test	
P value	0.1139
P value summary	ns
Significantly different (P < 0.05)?	No
One- or two-tailed P value?	Two-tailed
t, df	t=1.628, df=30
How big is the difference?	
Mean of column A	51.56
Mean of column B	82.56
Difference between means (B - A) :	31.00 ± 19.04
95% confidence interval	-7.883 to 69.88
R squared (eta squared)	0.08119
F test to compare variances	
F, DFn, Dfd	1.926, 15, 15
P value	0.2159
P value summary	ns
Significantly different (P < 0.05)?	No
Data analyzed	
Sample size, column A	16
Sample size, column B	16

disintegration E-cadherin KD

Table Analyzed	Data 1
Column B	E-cadherin KD
vs.	vs.
Column A	control
Unpaired t test	
P value	0.0352
P value summary	*
Significantly different (P < 0.05)?	Yes

One- or two-tailed P value?	Two-tailed
t, df	t=2.530, df=8
How big is the difference?	
Mean of column A	14.76
Mean of column B	25.93
Difference between means (B - A) ±	11.17 ± 4.415
95% confidence interval	0.9900 to 21.35
R squared (eta squared)	0.4445
F test to compare variances	
F, DFn, Dfd	3.245, 4, 4
P value	0.2807
P value summary	ns
Significantly different (P < 0.05)?	No
Data analyzed	
Sample size, column A	5
Sample size, column B	5

E-cadherin OE disintegration

Table Analyzed	Data 1
Column B	E-cadherin OE
vs.	vs.
Column A	Control
Unpaired t test	
P value	0.8297
P value summary	ns
Significantly different (P < 0.05)?	No
One- or two-tailed P value?	Two-tailed
t, df	t=0.2296, df=4
How big is the difference?	
Mean of column A	45.64
Mean of column B	44.67
Difference between means (B - A) ±	-0.9734 ± 4.240
95% confidence interval	-12.75 to 10.80
R squared (eta squared)	0.01301
F test to compare variances	
F, DFn, Dfd	5.805, 2, 2
P value	0.2939
P value summary	ns
Significantly different (P < 0.05)?	No
Data analyzed	

Sample size, column A	3
Sample size, column B	3

q	DF
5.176	16
0.1236	16
6.473	16

q	DF
9.748	24
0.9587	24
9.319	24

q	DF
4.538	7
0.08018	7
4.32	7

q	DF
3.966	6
1.644	6
5.61	6

q	DF
3.754	52
0.6335	52
4.109	52

q	DF
4.154	8
1.072	8
4.918	8

q	DF
3.989	50
6.274	50
9.565	50
